# Antithrombotics in heart failure

**DOI:** 10.3325/cmj.2014.55.621

**Published:** 2014-12

**Authors:** Davor Miličić, Jure Samardžić, Mate Petričević

**Affiliations:** 1 University of Zagreb School of Medicine, Department for Cardiovascular Diseases, University Hospital Center Zagreb, Zagreb, Croatia; 2 Department for Cardiac Surgery, University Hospital Center Zagreb, Zagreb, Croatia

## Abstract

Heart failure is a common clinical condition associated with high morbidity and mortality rate despite significant improvements in pharmacotherapy and implementation of medical procedures. Patients with heart failure are at an increased risk of developing arterial and venous thrombosis, which contribute to the high rate of adverse events and fatal outcomes. Many heart failure patients routinely receive antithrombotic therapy due to the presence of a specific indication for its use, like ischemic heart disease or atrial fibrillation. However, there is no solid evidence to support the routine use of antithrombotic agents in all heart failure patients. This article reviews the evidence for using antithrombotic therapy in heart failure patients.

Heart failure (HF) is a complex clinical syndrome caused by a number of different disorders that impair the heart muscle’s ability to maintain adequate blood flow to meet the body's metabolic needs. Research and advances in managing HF so far have been mainly focused on improving the neurohormonal imbalance and assisting the myocardium to increase the cardiac output with different types of devices. Over the last couple of decades there have been considerable improvements in pharmacotherapy and medical procedures for treating HF, such as the introduction of angiotensin-converting enzyme inhibitors (ACEIs), angiotensin receptor blockers (ARBs), beta blockers, aldosterone antagonists, I_f_ channel blocker ivabradine, cardiac resynchronization therapy (CRT), and ventricular assist devices (VADs). However, HF is still a major public health issue associated with increased morbidity and mortality (1). Because of the broad spectrum of its clinical presentations, aging of the population, and different comorbidities and treatment options, HF treatment represents a considerable challenge. Patients with HF are at an increased risk of developing arterial and venous thrombosis, which contribute to the high rate of adverse events and fatal outcomes (2-5). Further research should clarify whether routine use of antithrombotic agents brings clinical benefit to all HF patients.

## Thromboembolic burden of HF

HF patients have been reported to have increased platelet reactivity, reduced platelet survival time, and increased mean platelet volume (6-8). They have also been shown to have increased concentrations of fibrinogen and D-dimer, whose levels correlate with HF severity (9). Postmortem studies have shown that in patients with HF acute coronary incident represents an underrated cause of death. In the Assessment Of Treatment With Lisinopril And Survival (ATLAS) trial, acute coronary finding on autopsy was present in 33% of patients classified as having died from sudden cardiac death (5). Furthermore, the Optimal Trial in Myocardial Infarction with Angiotensin II Antagonist Losartan (OPTIMAAL) (10) has shown that an acute myocardial infarction was present in 55% and 81% of autopsies of HF patients classified as having died from arrhythmia and HF progression, respectively. As only 19% of patients had undergone autopsy, it is possible that this number was even higher (10). The prevalence of acute coronary incidents in HF patients is probably underestimated due to several factors such as HF itself, myocardial hybernation, cardiac denervation, or silent ischemia with co-existing diabetes, which mask the appearance of acute coronary syndrome. HF is also a common cause of ischemic stroke (11). In the population-based Framingham Heart Study, the relative risk of stroke in HF patients compared to those without HF was 4.1 for men and 2.8 for women (12). HF patients also have a high rate of stroke recurrence and mortality after stroke (13,14). Besides this, HF is an independent predictor of venous thromboembolism (VTE) (2,15-17). Patients with left ventricular ejection fraction (LVEF) of 20%-44% and less than 20% had a 2.8 and 38.3 times higher risk of developing VTE, respectively, meaning that severity of HF increased the risk of VTE (17). An increased risk of VTE was present in hospitalized HF patients compared to patients without HF, with a relative risk of 2.15 for pulmonary embolism and 1.21 for VTE (15). Having this in mind, chronic antithrombotic therapy seems a very rational treatment for these patients. Limited data on HF patients with preserved ejection fraction suggest that their risk of stroke is similar to that in HF patients with reduced ejection fraction, but the risk of thromboembolism is uncertain (18,19).

## What do guidelines say?

In 2012 European Society of Cardiology (ESC) issued new guidelines for treating HF, which state that other than in patients who have atrial fibrillation (AF) there is no evidence that oral anticoagulants reduce mortality and morbidity compared to placebo or aspirin in both HF with reduced and with preserved ejection fraction (1). Joint guidelines of American College of Cardiology Foundation (ACCF) and American Heart Association (AHA) for the management of HF from 2013 also do not recommend chronic anticoagulation therapy for patients without AF, a prior thromboembolic event, or an intraventricular thrombus (20). They also state that there is no evidence to use antiplatelet therapy in patients with HF in the absence of a specific indication (20). Similar recommendations have also been issued by the Joint Consensus Document from the ESC Heart Failure Association and the ESC Working Group on Thrombosis in 2012 (21) and the 2012 American College of Chest Physicians guidelines (22).

## Pathophysiology of venoarterial thrombosis in HF

Among risk factors for thrombotic events in HF are all three segments of Virchow's triad (blood stasis, blood vessel dysfunction, blood hypercoagulability). Reduction in blood flow and increase in the chance of intracardiac thrombus formation are caused by myocardial contractility dysfunction and enlargement of heart chambers (23). Abnormalities of the vessel walls in HF failure are caused by neurohormonal activation, inflammation, and endothelial dysfunction. HF impairs nitric oxide synthesis, which results in oxidative stress and promotes monocyte and platelet adhesion to the endothelium (24). Endothelial dysfunction in both arterial and venous system is a result of the distension of vessel wall and hypoxia. It is also enhanced by the increase in von Willebrandt factor concentration, soluble thrombomodulin concentration, and E-selectin levels (23,25,26). Activation of the sympathetic nervous system and of the renin-angiotensin-aldosterone system also plays a role in the enhanced thrombogenesis in HF as they increase β-thromboglobulin, platelet surface P-selectin, and plasminogen activator inhibitor-1 concentration levels, and thus, increase platelet aggregation and reduce fibrinolysis (25,26). Furthermore, low output and blood stasis contribute to the reduced renal clearance of procoagulation factors (27).

On the other hand, hemostatic disorder in HF patients might be also caused by comorbidities and medication. Patients with severe right HF can also develop congestive hepatopathy with impaired hepatic synthesis of coagulation factors II, V, VII, IX, and X, causing international normalized ratio (INR) prolongation and bleeding diathesis.

## Studies on antithrombotic agents in HF in sinus rhythm

### Retrospective observational analyses

Aspirin is the most widely used antithrombotic drug in HF patients in sinus rhythm, as ischemic heart disease is the most common condition that causes myocardial dysfunction (28). As there is very little solid evidence that long-term aspirin usage brings clinical benefit in both primary and secondary prevention of vascular events, its use in the setting of HF with sinus rhythm is questionable. Furthermore, aspirin in HF patients has never been directly compared against placebo in a randomized trial. At present there is no sufficient evidence to support the use of aspirin in all HF patients. The potential harm of aspirin use in HF patients lies predominately in its possible interaction with ACEIs, as observed in the post-hoc analysis of the Study Of Left Ventricular Dysfunction (SOLVD), where aspirin was found to decrease the survival benefit of ACEIs (29). This interaction is believed to be caused by aspirin’s effect on prostaglandin synthesis in the vessel wall (30,31). A systematic overview of data for 22,060 patients (mainly with known vascular disease) from six long-term randomized trials of ACEIs found no solid evidence that benefit of ACEI therapy was reduced when it was added to aspirin (32).

A possible interaction between aspirin and beta blockers was addressed in a retrospective analysis of 293 patients enrolled in a randomized, placebo-controlled trial of carvedilol. Among patients receiving carvedilol, those on aspirin therapy had less improvement in LVEF compared to aspirin nonusers and this effect was dose related. The mechanism behind this interaction is unknown (33).

The second most utilized antiplatelet agent today – clopidogrel, a second generation thienopiridine that does not inhibit prostaglandin production, was found to be a better antiplatelet option in HF patients than aspirin. This was shown by direct comparison in small randomized trials demonstrating that patients on clopidogrel had lower values of natriuretic peptides, better arterial function properties, and higher oxygen uptake during exercise testing (34-37). Bonde et al showed in a registry analysis of 50,000 patients hospitalized for the first myocardial infarction who were not treated with percutaneous coronary intervention that patients without HF had similar survival with or without clopidogrel, while patients with HF on clopidogrel had a significantly better survival compared to HF patients without clopidogrel (38). There is a lack of data on HF patients treated with newer, more potent antiplatelet agents – prasugrel and ticagrelor, as they have not been adequately tested in HF patients yet.

Analyses of the Spanish REDINSOR registry, which included 2263 HF patients with left ventricular ejection fraction ≤35% and sinus rhythm without other anticoagulation indications, showed no significant difference in total mortality and stroke incidence between patients who received anticoagulants (26%) and those who did not. Multivariate analysis, however, showed a decreased incidence in the combined endpoint of cardiovascular events (death, heart transplantation, coronary revascularization, hospitalization) in patients receiving anticoagulant therapy (39). Newer, available oral anticoagulants such as dabigatran – a direct thrombin inhibitor, or apixaban and rivaroxaban – factor Xa inhibitors, have not been specifically tested in patients with HF, especially in those in sinus rhythm.

### Important randomized controlled trials on antithrombotic agents in HF patients in sinus rhythm

Warfarin/Aspirin Study in Heart Failure (WASH) was a randomized study with 279 enrolled patients assigned to three groups – warfarin with target INR 2.5, aspirin 300 mg/d, and a group with no antithrombotic therapy. Mean follow-up period was 27 months. No difference was found in the primary endpoint (death, nonfatal myocardial infarction [MI], nonfatal stroke) between study groups. Patients on aspirin were twice as likely to be hospitalized due to cardiovascular cause or die during the first 12 months of follow-up compared to patients on warfarin. Around 75% of patients were male, 60% had an ischemic heart disease, and 4%-7% had atrial fibrillation (40).

Heart failure Long-term Antithrombotic study (HELAS) was prematurely stopped due to slow enrollment after having enrolled 197 patients out of the planned 6500. Patients with ischemic heart disease (n = 115) were randomized to 325 mg/d aspirin or warfarin (target INR 2.5-3.0), while 82 patients without ischemic heart disease were randomized to placebo or warfarin group (target INR 2.5-3.0). Patients were followed for a mean of 18.5 to 21.9 months. The sample was too small to detect any differences between the study groups in the primary composite endpoint and its components – non fatal MI, stroke, death, peripheral embolism, pulmonary embolism, rehospitalization, and HF worsening (41).

Warfarin and Antiplatelet Therapy in Chronic Heart Failure (WATCH) trial recruited 1587 patients with LVEF<35% and sinus rhythm. Patients were randomized to receive 162 mg/d aspirin, 75/d clopidogrel (double-blind fashion), and open label warfarin (target INR 2.5-3.0). Mean follow-up period was 1.9 years. There was no difference in composite primary endpoints (death, nonfatal MI, nonfatal stroke) between the study arms. Warfarin was associated with a reduced rate of stroke, but also with a higher rate of bleeding events compared only with clopidogrel group, but not with aspirin group. This trial did not show that warfarin or clopidogrel were superior to aspirin in HF patients in sinus rhythm (42).

Warfarin vs Aspirin in patients with Reduced Cardiac Ejection Fraction (WARCEF) trial was a large, randomized, double-blind, double-dummy trial that evaluated the efficacy of 325 mg/d aspirin vs warfarin with a target INR of 2 to 3.5. The study enrolled 2305 patients and only 43% had evidence of underlying ischemic heart disease. Mean follow-up was 3.5 years (range, 1-6 years). Compared to aspirin, warfarin did not significantly reduce the rate of the primary outcome (composite of ischemic stroke, intracerebral bleeding, and all cause mortality). A time-varying analysis showed a decreasing hazard ratio in warfarin group compared to aspirin and a significant benefit by the fourth year of follow-up. Warfarin group also had a 48% lower rate of ischemic stroke, but at the cost of significantly increased rate of major and minor bleeding events. Due to the low rate of ischemic stroke compared to bleeding events, the benefit of warfarin over aspirin in all HF patients was not demonstrated. Furthermore, patients on warfarin had a higher risk for hospitalization as opposed to WASH and WATCH study, where patients on aspirin had more hospitalizations. WARCEF is so far the largest randomized trial to address the usefulness of antithrombotic agents in HF in sinus rhythm (43). A subgroup analysis of WARCEF showed that in patients under 60 years, warfarin improved outcomes over aspirin with or without inclusion of major hemorrhage. For patients ≥60 years, there was no difference, but the aspirin group had significantly better outcomes when major hemorrhage was included (44).

## Antithrombotic agents in acute HF

Acute decompensated HF further increases the risk for thromboembolism (45) and these patients should be receiving anticoagulation therapy as VTE prophylaxis until compensated and mobilized. Whether antiplatelet therapy could improve prognosis in acute HF is not clear. Kozdag et al showed on 580 patients discharged from hospital after acute decompensated HF (63% with coronary artery disease) that patients on clopidogrel had statistically significant decrease in cardiac mortality compared to patients who did not take clopidogrel (46).

## Future directions and perspectives

Due to population aging and increased survival of patients with cardiovascular diseases, prevalence of HF will continue to increase (11). The debate about how antithrombotic agents can improve outcomes of HF patients in sinus rhythm is still open. It would certainly be interesting to test the efficacy of antithrombotic therapy not only on top of, but also against contemporary optimal medical therapy for HF. However, for ethical reasons this comparison will probably never be done.

Antithrombotic therapy in HF is intensively investigated by ongoing trials. A study (NCT01534026) investigating the effect of withdrawing aspirin compared to its use will address the need to prescribe aspirin to non-ischemic HF patients in sinus rhythm (47). Clopidogrel Vs Aspirin in Chronic Heart Failure (CACHE), a non-blinded, large randomized controlled trial (ISRCTN22153967) plans to enroll 3000 patients with HF in sinus rhythm and investigate whether aspirin 75 mg/d, compared to clopidogrel 75 mg/d, increases mortality and the number of HF hospitalizations, and reduces the quality of life. The planned mean duration of follow-up is 3.8 years and the study is expected to end in August 2016 (48). COMMANDER HF trial (NCT01877915) is planned to assess the effectiveness and safety of rivaroxaban compared to placebo in reducing the risk of death, myocardial infarction, or stroke in 5000 HF patients with significant coronary artery disease following a recent hospitalization for HF exacerbation (49).

## Conclusion

To date, there has been no solid evidence to support the use of antithrombotic agents in all HF patients despite the fact that HF is associated with hypercoagulability and increased platelet activation. Treating chronic HF patients with antithrombotic agents without a proper indication such as recent coronary stenting, atrial fibrillation, implanted prosthetic valve, or ventricular assist device, is controversial, and routine use of these agents is not recommended for now in these patients. As HF patients are a heterogenous population, future trials should be carefully designed. The risk-benefit ratio in HF patients in sinus rhythm could be improved using platelet function testing and thromboelastometry. Also, adequately designed, large-scale randomized placebo-controlled trials involving new antiplatelet and new oral anticoagulant agents are also warranted to clarify the necessity of giving antithrombotic agents to all HF patients.
